# Lignin Resists High-Intensity Electron Beam Irradiation

**DOI:** 10.1021/acs.biomac.1c00926

**Published:** 2021-09-10

**Authors:** Oliver Sarosi, Irina Sulaeva, Elisabeth Fitz, Ivan Sumerskii, Markus Bacher, Antje Potthast

**Affiliations:** † Kompetenzzentrum Holz GmbH, Altenbergerstraße 69, A-4040 Linz, Austria; ‡ Institute of Chemistry of Renewable Resources, Department of Chemistry, University of Natural Resources and Life Sciences, Konrad-Lorenz-Straße 24, A-3430 Tulln, Austria

## Abstract

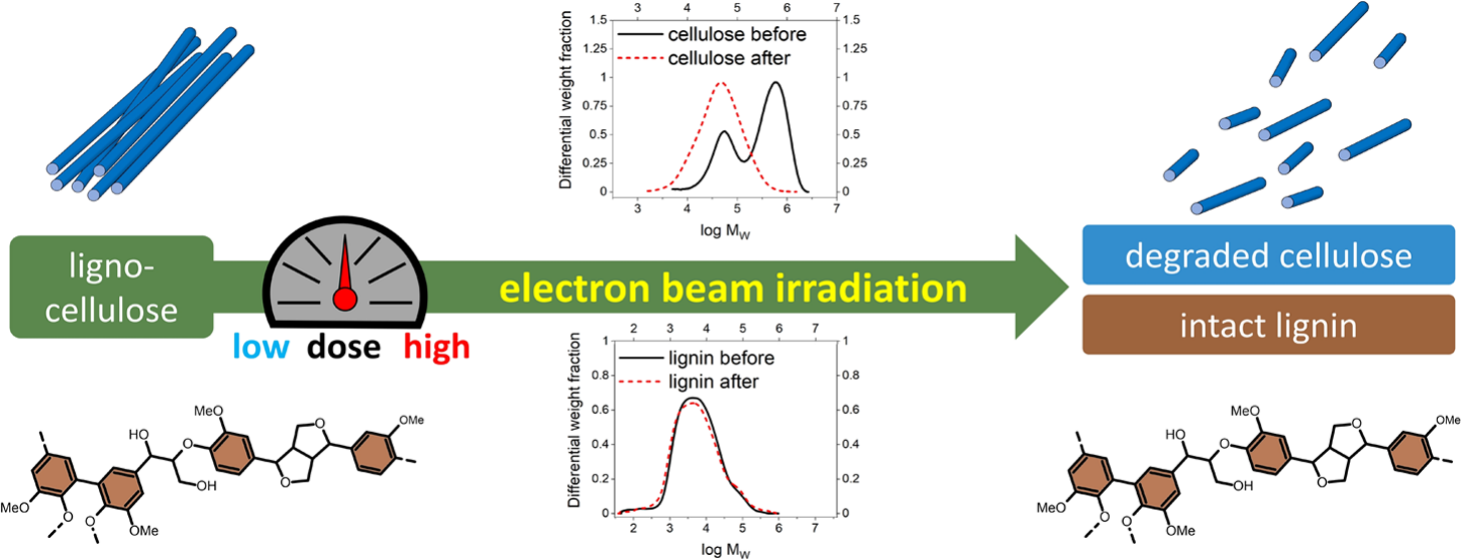

The electron beam irradiation (EBI) of native lignin has received little attention. Thus, its potential use in lignin-based biorefineries is not fully understood. EBI was applied to selected lignin samples and the structural and chemical changes were analyzed, revealing the suitability, limitations, and potential purpose of EBI in wood biorefineries. Isolated milled wood, kraft, and sulfite lignin from beech and eucalyptus were subjected to up to 200 kGy of irradiation. The analysis included gel permeation chromatography for molar masses, heteronuclear single quantum coherence (HSQC)- and ^31^P NMR and headspace gas chromatography-mass spectrometry for functional groups, and thermogravimetric analysis for thermal stability. Most samples resisted irradiation. Subtle changes occurred in the molecular weight distribution and thermal stability of milled wood lignin. EBI was found to be a suitable pretreatment method for woody biomass if the avoidance of lignin condensation and chemical modification is a high priority.

## Introduction

Electron beam irradiation (EBI) facilitates the controlled degradation of lignocellulosic feedstock, and thus it may be incorporated in lignin-based biorefineries. First, the structure and properties of lignin and the effect of EBI must be considered. Lignin is a heterogeneous polymer, consisting of covalently linked phenolic units, which allow it to stabilize radicals.^1^ Previous studies described the important chemical and structural properties of lignin with regard to its radical scavenging activity (RSA).^2^ It was found that the free nonetherified hydroxyl groups on the aromatic ring are essential for lignin’s antioxidative effect because they form stable quinoid structures through phenoxy radical intermediates.^3^ Furthermore, an extended π-electron system helps to improve the RSA. In contrast, strong methylation and oxidation of lignin lower the antioxidative effect. Consequently, chemical treatments, such as kraft and sulfite pulping, which significantly alter the chemical structure, change the irradiation resistance of native lignin. In a comparative study in which lignin model compounds were irradiated with EBI, the β-aryl ether bond was more susceptible to radiolytic cleavage when the compound was phenolic, i.e., less methoxylated and vice versa.^4^ This effect is highly dependent on the presence or the absence of H-abstracting species, such as peroxides and moisture content.^5^ The explanation lies within the facilitated abstraction of a phenolic hydrogen radical followed by resonance distribution and radiolytic cleavage. When irradiated with an electron dose of up to 90 kGy, the number of peroxy radicals in kraft lignin increased.^6^ This was indicative of degradation and demethylation reactions. Through Fourier transform infrared spectroscopy, an increase in the number of conjugated structures was apparent, thus suggesting polycondensation of the degraded lignin structures. At very high irradiation doses (200–1000 kGy), lignin, unlike cellulose, exhibits condensation, which appears as a small shoulder in the high molecular region of the molecular weight distribution (MWD).^7,8^ Yet, lignin degradation by bond cleavage is the predominant reaction. Another study applied γ-irradiation at a dose of 1200 kGy to kraft lignin and found a reduction in the weighted average molecular mass (*M*_w_) of up to 24% in gel permeation chromatography (GPC).^9^ Compared to lignin, cellulose is heavily degraded by EBI at doses below 100 kGy.^10^ Thus, EBI may facilitate the isolation of lignin from biomass while retaining its original structure. Upon irradiation of lignocellulosic materials, radicals are generated in cellulose, hemicellulose, and lignin, which leads to their decomposition by scission of chemical bonds or oxidation.^11^ Low doses, 1–25 kGy, can be used to moderately decrease the degree of polymerization (DP) of cellulose during the production of dissolving pulp.^12,13^ At higher doses, 50–1000 kGy, EBI strongly decreases the average length of the cellulose chain, thereby facilitating saccharification in fermentation.^8,14−17^ However, because of diminishing returns in terms of saccharification yields, high operation costs, and throughput limitations, a 100–200 kGy dose is more appropriate for biomass pretreatment.^18−20^ The ionizing irradiation dose may thus be chosen to facilitate the selective degradation of the cellulosic components of woody biomass without a measurable effect on lignin. The chemical and structural changes in lignin, especially at doses of 100–200 kGy, have been rarely studied in detail.^10,21−23^ Previous studies on the antioxidative effects of lignin and the analysis of associated functional groups have focused on free hydroxyl groups; thus, changes in other parts of the molecules were overlooked. The current study elucidates the chemical and structural changes in milled wood lignin (MWL) from beech (*Fagus sylvatica*) and eucalyptus (*Eucalyptus grandis × urophylla*) after EBI at a dose of up to 200 kGy. For a further point of reference, technical lignin from beech (*F. sylvatica*) and eucalyptus (*Eucalyptus globulus*) was included.

## Experimental Section

### Lignin

*E. globulus* kraft and *F. sylvatica* sulfite lignin were isolated from spent cooking liquors by adsorption to an Amberlite XAD-7 ion-exchange resin, followed by desorption with ethanol. Both samples were kindly contributed as a dry powder by the chemistry department of the University of Natural Resources and Life Sciences, Vienna. Three MWL samples were produced: two from different variants of *F. sylvatica* and one from *E. grandis × urophylla*. The wood samples, in addition to the general analysis data, were received as chips from Lenzing AG. The procedure was an adapted variant of the isolation and purification technique described in an earlier study.^24^ First, the wood was dried and milled to a particle size of 35 mesh. To remove the extractives, the wood meal was extracted for 24 h in a Soxhlet extractor. A mixture of acetone and water (1:1) at a ratio of 10 mL g^–1^ was used. The wood was then milled using a Retsch PM100 (Haan, Germany) rotary ball mill with a 500 mL milling jar and eight balls with 20 mm diameter. The ball milling equipment was made out of ZrO_2_ and milling was performed at 250 rpm for 14 h with a 30 min break after every 15 min of milling to allow cooling. Extraction of the MWL was performed by swirling the wood powder in a mixture of 1,4-dioxane and water (9:1) at a ratio of 10 mL g^–1^ of the wood powder. The extraction was performed for 72 h and the solvent was exchanged every 24 h. All fractions were combined and the solvent was evaporated while the temperature was kept below 35 °C at all times. The crude MWL was purified by dissolution in the least possible amount of a 1:1 mixture of glacial acetic acid and water, centrifugation to remove insoluble solids, and dropwise precipitation in a 10-fold excess of swirling water. After centrifugation, the solids were washed and centrifuged three times with fresh water and transferred to a round flask using a mixture of acetone and water (9:1). To remove residual acetic acid, the acetone was evaporated, and fresh acetone/water was added for a total of four evaporation cycles. To remove carbohydrates, the crude MWL was dissolved in the least possible amount of a 2:1 mixture of 1,2-dichloroethane and ethanol. The solution was centrifuged to precipitate the lignin–carbohydrate complexes and the supernatant was used for further purification. The MWL was precipitated dropwise into swirling diethyl ether and centrifuged to remove the solvents. The solid MWL was washed four times with diethyl ether. Dissolution in 1,2-dichloroethane/ethanol and the ether precipitation was repeated four times. After the last iteration, the purified MWL was washed with petrol ether, dried by rotary evaporation, and finally freeze-dried under high vacuum. Tables 1 and 2 list the samples that were used in this study, including the wood source and isolation methods.

**Table 1:** Lignin Samples, Their Wood Sources, and Their Isolation Origins^a^

sample name	wood source	origin
MWL A	*F. sylvatica*	Björkman lignin from short-storage beech
MWL B	*F. sylvatica*	Björkman lignin from long-storage beech
MWL C	*E. grandis × urophylla*	Björkman lignin from eucalyptus
KL	*E. globulus*	Kraft lignin isolated from spent cooking liquor using ion-exchange resin
LS	*F. sylvatica*	Lignosulfonate isolated from spent cooking liquor using ion-exchange resin

a For MWL B, dissolution in 1,2-dichloroethane and ethanol was substituted once using methanol as a solvent. However, MWL B was mostly insoluble in methanol. In addition, it congealed upon methanol infusion and exhibited a dark olive tint. Subsequently, ∼80% of MWL B was also insoluble in 1,2,-dichloroethane and ethanol, potentially indicating polymerization that occurred in methanol.

**Table 2:** Carbohydrate Content of the Lignin Samples as Measured by Total Hydrolysis and HPLC^a^

sample	glucan (%)	xylan (%)	mannan (%)	arabinan (%)	rhamnan (%)	galactan (%)	sum (%)
MWL A	0.3	4.4	<0.1	0.3	0.2	0.3	5.5
MWL B	0.3	1.3	<0.1	0.1	0.1	0.2	2.0
KL	0.2	1.0	<0.1	0.1	<0.1	0.2	1.4
LS	0.1	0.2	0.1	<0.1	<0.1	<0.1	0.4

a MWL C could not be included because of material constraints.

### Irradiation

EBI was performed at the Mediscan GmbH & Co KG (Kremsmünster, Austria) using a Rhodotron TT100-IBA-X electron accelerator with a beam power of 10 MeV, according to EN ISO 13485 and ISO 11137. For each sample, 120–600 mg of the dry lignin powder was poured in 1.5 mL microreaction tubes and attached to a cardboard sheet. Irradiation intensity was set to (and verified as) 1.25 kGy (1.30 kGy), 2.5 kGy (2.52 kGy), 5.0 kGy (5.2 kGy), 10.0 kGy (10.3 kGy), 50.0 kGy (50.2 kGy), 100 kGy (101.3 kGy), or 200 kGy (202.0 kGy). For the latter two doses, the samples were irradiated by multiple passes at 50 kGy on alternating sides. The irradiation dose was verified by attaching test strips to every batch for evaluation with a dosimeter. For the MWL samples, only the 200 kGy dose was performed. Irradiated lignin was used for analysis without further purification.

### Lignin Analysis

The molecular weight distribution of the lignin samples was analyzed using GPC in accordance with a method described previously.^25^ The samples were dissolved in dimethyl sulfoxide (DMSO)/LiBr (0.5% w/v), filtered through a 0.45 μm syringe filter, and injected into a GPC system, which was constituted of a precolumn and three columns (Agilent PolarGel M), a refractive index detector, and a multiangle light scattering (MALS) detector with a 785 nm laser.

The quantification of methoxy groups in lignin was performed with the headspace isotope dilution method described previously.^26^ The method relies on the addition of the isotopically labeled internal standards 4-(methoxy-*d*_3_)-benzoic acid and 4(ethoxy-*d*_5_)-benzoic acid, followed by standard hydroiodic acid cleavage of the methoxyl groups, and finally their analysis by headspace gas chromatography-mass spectrometry (GC-MS).

For functional group analysis, ^31^P- and heteronuclear single quantum coherence (HSQC)-NMR were employed using a Bruker Advance II 400 or a Bruker Advance III HD 400 (Bruker, Billerica, MA). The exact device settings were adjusted as described previously.^27,28^ For the determination of hydroxyl groups in ^31^P NMR, the lignin samples were dissolved in CDCl_3_/pyridine (1:1.6). As an internal standard, 200 μL of *N*-hydroxy-5-norbornene-2,3-dicarboxylic acid imide stock solution (0.02 mol mL^–1^) and an NMR relaxation agent, chromium acetylacetonate [Cr(acac)_3_; 5 mg mL^–1^], were added. Finally, the samples were phosphitylated by the addition of 100 μL of 2-chloro-4,4,5,5-tetramethyl-1,3,2-dioxaphospholane under moisture exclusion and shaking for 1 h at room temperature. For HSQC analysis, roughly 50 mg of a dry lignin sample was dissolved in 0.6 mL of DMSO-*d*_6_ and used directly for analysis. The measurement data was evaluated using MNova 14.2.0-26256. Signal annotation was pursued with reference to the existing literature.^27−35^ For the volume integration of cross peaks, an oval integration area mode was used. Integrals were placed at a single intensity level for each sample spectrum. The integral regions of the reference and irradiated versions were identical. Where possible, the overlapping signals were integrated separately. The standard deviation depends on the type of the OH group analyzed; thus, it was tested with Indulin AT lignin (*N* = 5). It ranged from carboxyl groups’ relative standard deviation (RSD) = 2.24%, aliphatic hydroxyls RSD = 1.76%, syringyl and condensed RSD = 0.97%, guaiacyl and catechol RSD = 0.68%, aromatic hydroxyls RSD = 0.74%, and total hydroxyls RSD = 0.80%.

Thermogravimetric analysis (TGA) was performed by placing 2–5 mg of the dry lignin powder in the weighing tray of a Pyris 1 thermogravimetric analyzer (Perkin-Elmer Inc., Waltham, MA). The heating rate was set to 10 °C min^–1^ and a range of 25–550 °C was recorded. The on-set points for the start of thermal degradation were assessed by annealing linear lines to the respective sections of the curve.

The residual carbohydrate content was measured by the total hydrolysis of a 100 mg sample using sulfuric acid and subsequent high-performance liquid chromatography (HPLC). The analysis system consisted of a DIONEX ICS 5000+ HPLC (Thermo Fisher Scientific Inc., Waltham, MA), a CarboPac SA10 anion exchange column, and a pulsed amperometric detector. An injection volume of 10 μL and a flow rate of 1 mL min^–1^ were used.

## Results and Discussion

MWL from *F. sylvatica* was included twice. The difference between the two samples was the storage time for the logs in the lumber yard before isolation. During the isolation of MWL B, a few steps were altered. This might have led to greater property differences than those naturally present in the wood. This will be discussed where necessary.

### Molecular Weight

**Figure 1:**
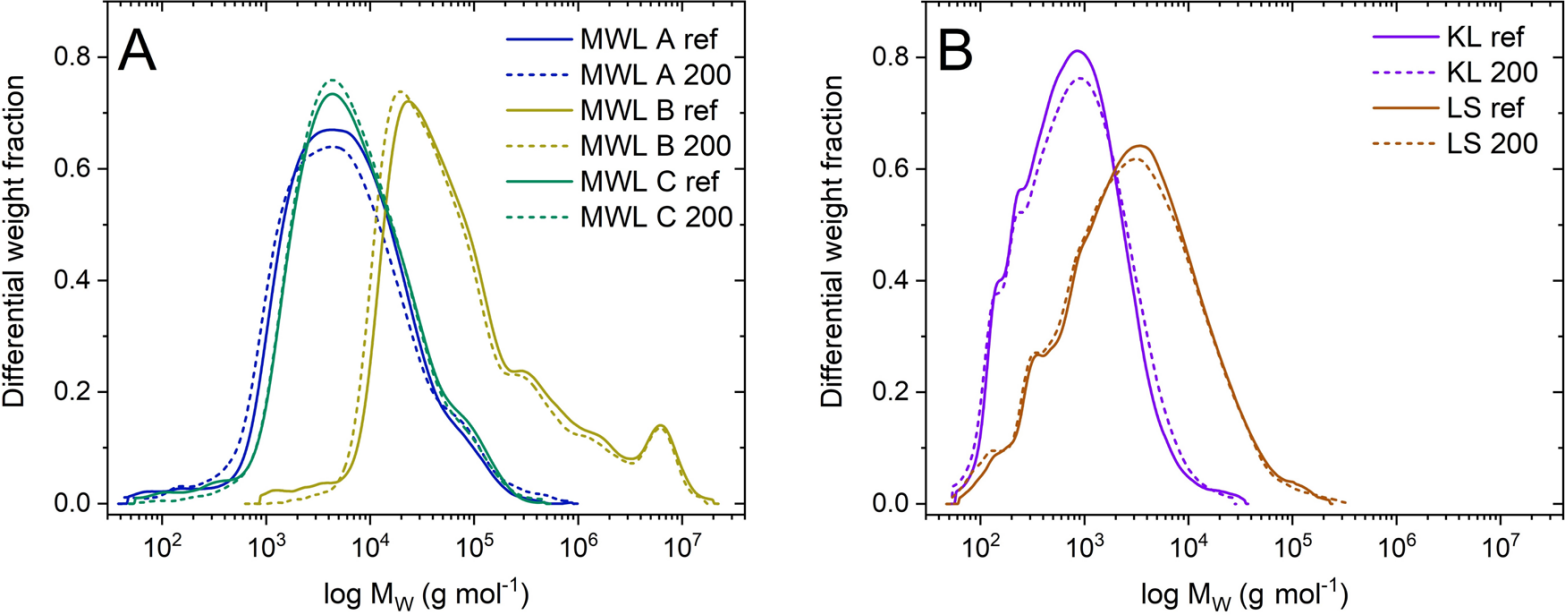
Molecular weight distribution of (A) milled wood lignin and (B) technical lignin from kraft and sulfite processes before and after irradiation at a dose of 200 kGy.

The GPC measurements revealed subtle changes in the molar masses of all tested lignin samples upon irradiation. Marginal changes were observed in the statistical moments (see the Supporting Information) with MWL A and MWL B showing a 23% increase and 12% decrease in *M*_w_, respectively. More importantly, a visual comparison of the MWD (Figure 1) showed no significant differences in lignin in all cases. This gives rise to high stability of the lignin structure under EBI at irradiated doses up to 200 kGy. In comparison, the molar mass of cellulose is heavily reduced under equal conditions.^10^ Since cellulose is a linear polymer, every single cleavage of the glycosidic bond will split the cellulose chain, thus reducing the molar mass. The key to the preservation of lignin’s molar mass is found in its structure. Lignin is composed of a branched three-dimensional (3D)-network, which is not split upon cleavage of single or even multiple interunit linkages. As seen for cellulose, EBI statistically favors the attack of large molecules because of their increased surface area. This was not observed here. MWL B shows this most clearly with a distinct group of highly polymerized species around 6 × 10^6^ g mol^–1^, which remained virtually unchanged after EBI. The reason for the occurrence of this distinct peak remained unclear. It may be associated with the differential treatment of MWL B during isolation.

Another factor for the resistance of lignin’s molar mass is the level-off DP. It describes the lower limit at which a given polymer can no longer be degraded to a measurable degree by a given treatment.^36^ In the case of EBI, a level-off DP starts to occur when the ionization events are distributed across a high number of small particles, i.e., if the molar mass is already at a very low level. This is the case for both technical lignins, KL and SL, and may also partially apply for MWL A and MWL C. An additional series of irradiation experiments were conducted, using a stepwise dose increase on KL and SL, to exclude the oversight of local minima or maxima in *M*_w_. It revealed no additional information (data not shown).

Previous studies have reported the degradation of softwood kraft lignin at a dose up to 10 kGy and cross-linking at a dose up to 50 kGy, with *M*_w_ variations of −10 and +15%, respectively.^37^ In another study, the application of very high doses up to 1200 kGy to alkali lignin led to a reduction in *M*_w_ from 17.8 to 13.5 kDa (−24%). However, doses up to 600 kGy produced only minimal changes.^9^ Another study investigated the combined use of EBI at 100 kGy and steam explosion as wood pretreatment.^8^ The EBI-induced changes in the MWD were minimal and appeared to be less severe than those produced by steam explosion. A common trend in these studies was the finding of a smaller effect of ionizing irradiation on the molar mass when the samples were fully dried. This was attributed to the lack of secondary reactions with solvent radicals.

### Methoxy Group Content

The methoxy group content was determined with a method that relies on headspace isotope dilution GC-MS, the protocol that provides the most reliable data.^26^ The data for the investigated lignin before and after irradiation are summarized in Table 3. The findings of the current study do not support those of previous studies, which indicated a positive correlation between free phenolic groups and lignin degradation upon irradiation.^38^ The changes in the methoxy group content upon irradiation were less than the respective RSD for each sample. The exception was MWL B, which exhibited a significant increase of 7.9%. Demethoxylation was not observed. The reason could be the lack of water and the necessary free hydroperoxyl radicals.^5,39^ In the case of MWL B, the origin and underlying mechanism for the increase in methoxy group content is unclear. Methylation requires the presence of an activated methyl group donor that is reactive toward OH groups. Methylation performed by enzymes, such as caffeoyl-CoA *O*-methyltransferase, during lignin biosynthesis relies on S-adenosyl methionine as the methyl group donor.^40^ In organic synthesis, dimethyl sulfate or methyl chloride can be used to methylate hydroxyl groups under alkaline conditions. However, the generation of methenium ions or their precursors in lignin upon irradiation has not yet been described. The possible precursors include solvent methanol that was not fully removed by lyophilization or formaldehyde. It can be formed by the reverse aldol reaction of the γ carbon of the aliphatic lignin side chain.^41,42^ The radical recombination reaction of a lignin radical and a methoxy radical presents a possible pathway for methylation. Whether this pathway occurs in the given quantities or why it would occur only in MWL B is unclear.

**Table 3:** Methoxy Group Content of Lignin before and after Irradiation with Accelerated Electrons at a Dose of 200 kGy^a^

sample	OMe reference (mmol g^–1^)	OMe 200 kGy (mmol g^–1^)	OMe difference (Δ%)
MWL A	6.0 ± 0.2	6.1 ± 0.2	+1.7
MWL B	6.3 ± 0.3	6.8 ± 0.3	+7.9
MWL C	5.2 ± 0.2	5.2 ± 0.2	± 0.0
KL	6.3 ± 0.3	6.2 ± 0.2	–1.6
LS	4.9 ± 0.2	4.9 ± 0.2	± 0.0

a Error range corresponds to the calculated relative standard deviation of up to <4%.

### HSQC-NMR

**Figure 2:**
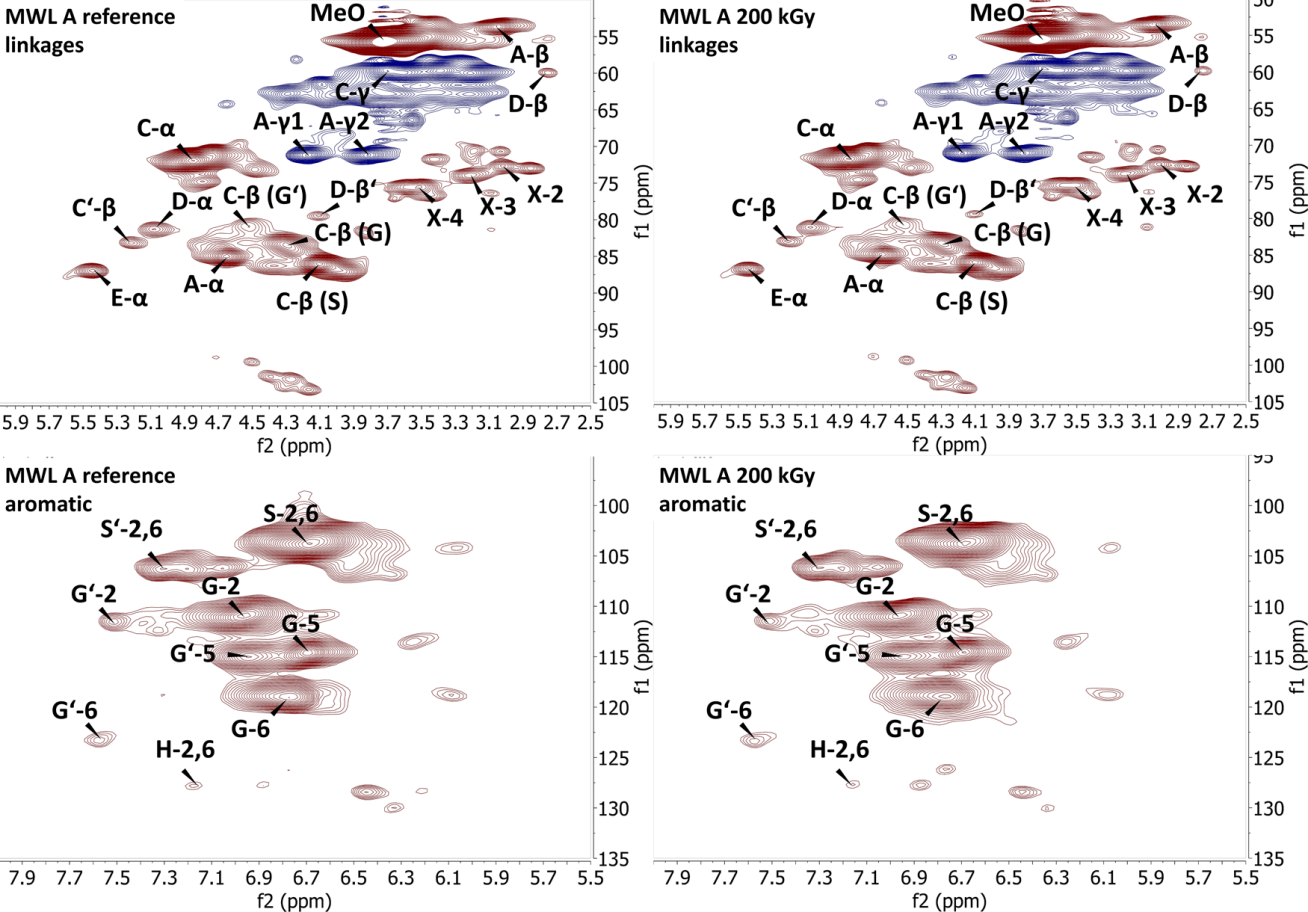
HSQC-NMR sections of MWL A before and after irradiation at 200 kGy (for acronyms, see the Supporting Information).

Semiquantitative HSQC-NMR techniques facilitated the comparison of the reference and irradiated lignin to identify the chemical changes. Several approaches were applied to normalize the 2D-NMR signals. For the first normalization approach, a cluster of aromatic signals that are a representative of all C9 units were used as an internal standard.^34^ This method delivered the most reliable results. Aromatic groups are considered to be very stable under EBI at the given doses.^9^ The reference signal for normalization was calculated as follows
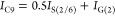
1where *I*_S(2/6)_ is the sum of integrals of both S(2/6) and S′(2/6) and *I*_G(2)_ is the sum of the integrals of both G(2) and G′(2) (i.e., oxidized G-units). Integral normalization was calculated as follows
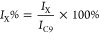
2where *I*_X_ is the integral of any given signal. For KL and LS, G(2) and G′(2) could not be integrated properly because of overlapping signals or low signal intensities. This might have limited the comparison of KL and LS to other lignin samples. However, the changes in each reference and irradiated sample were monitored. The results (for a detailed summary of normalized integrals, see the Supporting Information) indicate that EBI produced no significant changes in the characteristic bonds of any of the lignin samples. The signal of the more stable β–β bond and that of the more fragile β–O–4 bond did not decrease. The S/G ratio decreased slightly. The exception was KL, in which there was a slight increase. To increase the S/G ratio through irradiation, the protons on either the S(2/6) or S′(2/6) positions must be lost (e.g., through derivatization), or those on the G(2) and G′(2) positions must be liberated. The latter pathway is chemically implausible; however, the former may be caused by condensation reactions. Another possibility is related to the aforementioned methoxylation, which would have to occur on the aromatic ring of G-units. This would cause the NMR to be shifted outside the G(2)/G′(2) integration area or the formation of S(2/6)/S′(2/6) if methylation occurs at the 5′ position. However, this seems unlikely because an increase in the methoxy group content was not detected in the KL. In the MWL samples, the changes in the methoxy group content determined by semiquantitative HSQC-NMR diverged from the results of direct headspace-GC-MS (HS-GC-MS). In the case of technical lignin, the trends detected in both types of measurements aligned. However, GC-MS delivered more accurate values, albeit with insignificant changes. In the remaining HSQC signals, neither semiquantitative evaluation nor a direct comparison of the spectra revealed significant modifications (Figure 2; see the Supporting Information for all spectra and a list of acronyms). The presence or the absence of lignin–carbohydrate complexes (LCCs) was investigated in the HSQC-NMR spectra of the current study.^35^ The results did not support those of previous studies regarding the destruction of LCCs by ionizing irradiation.^38^ Only traces of benzyl ether LCC structures were found in the HSQC-NMR spectra of KL and LS. The magnitude of their cross peaks remained unchanged after EBI.

**Figure 3:**
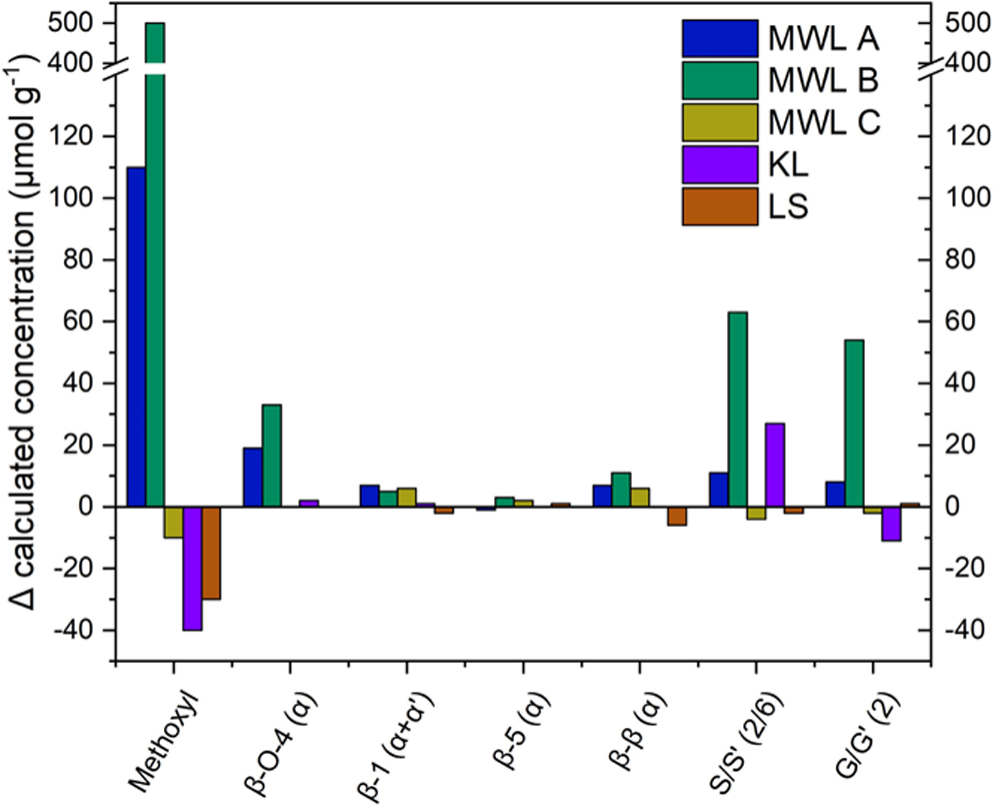
Calculated concentration differences in lignin bonds in irradiated and nonirradiated samples.

Another referencing method that used the measured methoxy group concentration as an external standard was chosen. The concentration of a given lignin HSQC signal, “X”, was calculated as follows
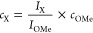
3where *I*_X_ is the integral of the given signal, *I*_OMe_ is the integral of the methoxyl group signal, and *c*_OMe_ is the measured methoxy group concentration from GC-MS. These calculations revealed concentration differences in the characteristic bonds of the untreated and irradiated lignin samples (Figure 3). The changes in the concentration were very minimal for all samples, considering the displayed concentration range, with no visible correlations. The trend of concentration increases was observed in MWL A and B. The reason might be the bias introduced by the measured differences in *c*_OMe_ of +1.7 and +7.9%.

### ^31^P NMR

At this point, the analysis results indicated that EBI at 200 kGy had little to no significant structural or chemical effect on lignin. Thus, ^31^P NMR was performed on two selected samples. Using a norbene-type internal standard, the concentrations of different species of OH groups were calculated (Table 4). The observed changes lie either below or barely above the RSD, indicating very minimal effects overall. As such, both samples showed a 2–4% increase in syringyl/condensed hydroxyl groups, indicating little condensation. Furthermore, a 10% increase in carboxyl groups was detected, suggesting oxidation, however, at an overall low level. Additionally, MWL A showed a 2.5% increase in aromatic hydroxyl groups through cleavage of ether bonds, which is also reflected in GPC data.

**Table 4:** Hydroxyl Group Concentration in Lignin Samples before and after Irradiation, as Measured by ^31^P NMR^a^

sample	aliphatic (mmol g^–1^)	syringyl and condensed (mmol g^–1^)	guaiacyl and catechol (mmol g^–1^)	carboxyl (mmol g^–1^)	aromatic (mmol g^–1^)	total (mmol g^–1^)
MWL A Ref	4.98 ± 0.09	0.469 ± 0.004	0.550 ± 0.003	0.120 ± 0.003	1.02 ± 0.01	6.00 ± 0.05
MWL A 200	4.99 ± 0.09	0.504 ± 0.004	0.573 ± 0.003	0.142 ± 0.003	1.08 ± 0.01	6.07 ± 0.05
MWL C Ref	3.58 ± 0.06	1.17 ± 0.01	1.26 ± 0.01	0.188 ± 0.004	2.43 ± 0.02	6.01 ± 0.05
MWL C 200	3.57 ± 0.06	1.19 ± 0.01	1.26 ± 0.01	0.207 ± 0.005	2.45 ± 0.02	6.02 ± 0.05

a For the respective relative standard deviation, see the Experimental Section.

### Thermogravimetric Analysis

**Figure 4:**
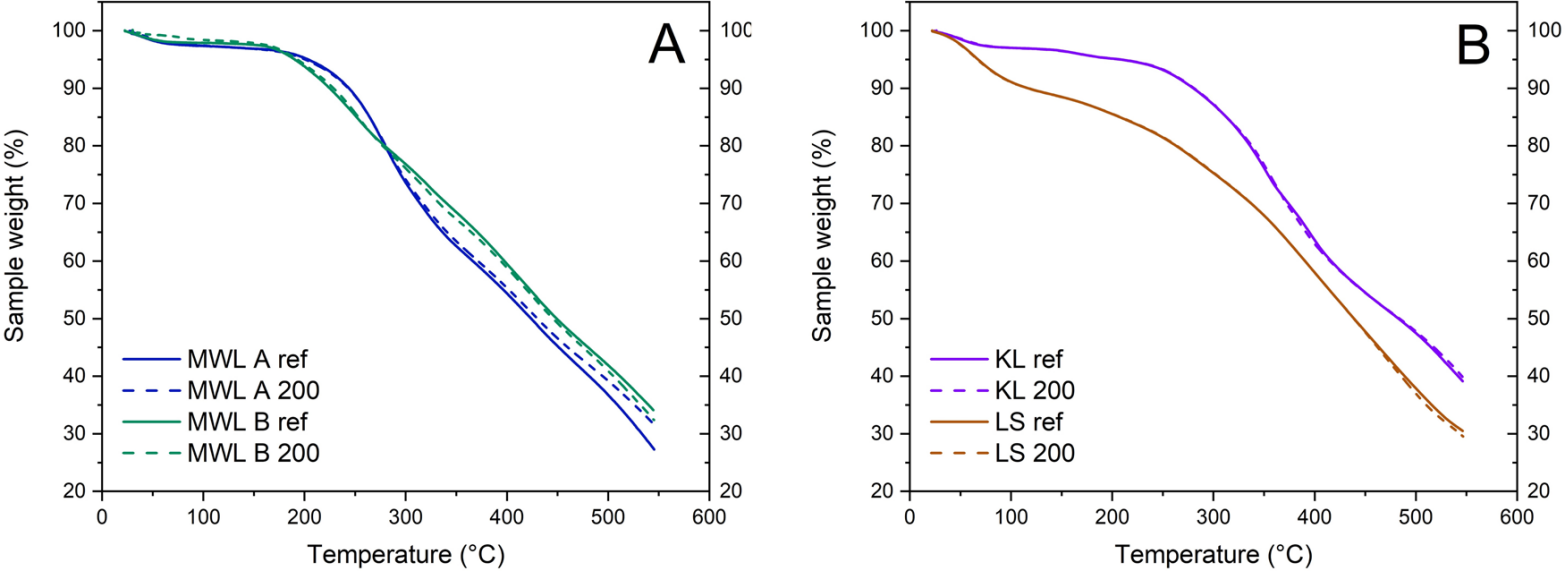
Thermogravimetric analysis of (A) milled wood lignin and (B) technical lignin samples before (solid lines) and after (dotted lines) irradiation at 200 kGy.

As is the case with radiation resistance, the thermal resistance depends on the structural and chemical composition of lignin. Thermogravimetric analysis (TGA) revealed the relatively strong stability of KL, moderate stability of MWL A and B, and lower stability of LS (Figure 4). There were major differences in the lignin types regarding the on-set point for thermal decomposition (Table 5).

**Table 5:** On-Set Points for Thermal Degradation of Lignin Samples during Thermogravimetric Analysis

sample	weight (%)	temperature (°C)
MWL A reference	95.8	228.6
MWL A 200 kGy	94.5	230.7
MWL B reference	97.7	186.6
MWL B 200 kGy	97.3	194.8
KL reference	93.3	286.5
KL 200 kGy	93.4	288.9
LS reference	N/A	N/A
LS 200 kGy	N/A	N/A

However, the differences between each reference and irradiated sample were more subtle. TGA indicated that there was virtually no difference in the technical lignin samples KL and LS before or after irradiation. For the MWL samples, the TGA curve of the irradiated variant was slightly displaced to the reference. MWL A displayed stronger degradation and MWL B exhibited weaker degradation during pyrolysis at 280–550 °C. This might be correlated with the changes in *M*_w_ (see the Supporting Information). A higher average molecular mass increased thermal resistance and vice versa. This is also expressed by the thermal degradation on-set points, which increased for MWL B by 8.2 °C. LS did not allow for on-set point analysis because its decomposition began before the complete evaporation of residual water. MWL C could not be included because of material depletion. Other researchers have found a negative offset of the thermal degradation profiles in alkali lignin after irradiation doses up to 1200 kGy.^9^ In a study that used more comparable irradiation doses, differential scanning calorimetry indicated an 18 °C decrease in the glass-transition temperature of kraft lignin after an irradiation dose of 90 kGy.^6^ In contrast, the results of TGA in the current study suggested that irradiation had a much less severe effect on the thermal stability of lignin.

## Conclusions

Milled wood, kraft, and sulfite lignin samples from beech and eucalyptus were subjected to EBI, and the effects were analyzed using GPC, HS-GC-MS, NMR, and TGA. Upon irradiation at a dose of 200 kGy, the samples remained mostly unaffected. This was attributed to their 3D structure, antioxidative properties, and radical scavenging activity. The MWD of MWL of beech from short-term log storage indicated moderate degradation. However, the long-term storage variant exhibited predominately lignin cross-linking. Either effect, cross-linking or degradation, was limited to the high molecular mass region because the changes in *M*_z_ were the highest, followed by those in *M*_w_. *M*_n_ remained almost constant. The thermal decomposition rate of the MWLs was correlated with the changes in the molecular mass upon irradiation; however, the difference was minimal. Demethoxylation, which was described in previous studies, was not observed. Several normalization techniques were used for the semiquantitative evaluation of HSQC-NMR results. However, neither revealed significant chemical changes in any of the tested samples. Similarly, the free OH group content measured by ^31^P NMR remained nearly constant between reference and irradiated lignin. The formation of minimal amounts of free phenolic hydroxyl and carboxylic acid groups was attributed to depolymerization and oxidation. The overall effect of EBI at 200 kGy on lignin was small. The majority of the properties of the investigated samples were retained. Previously, the effect of EBI at 200 kGy on cellulose pulps was proven to be much stronger, thereby leading to both degradation and backbone oxidation. In future work, this dual effect on cellulose and lignin may be applied to biorefinery concepts. EBI can be used for the pretreatment of woody biomass, prior to the chemical or enzymatic removal of (hemi)cellulosic components. EBI had almost no effect on the remaining lignin fraction; thus, the isolation of lignin in a close-to-native state may be facilitated.

## Supporting Information Available

Chemical composition of wood used for MWL isolation, calculated statistical moments from GPC of all samples before and after irradiation, HSQC-NMR spectra, tables for chemical shifts, assignment of cross peaks, and volume integrals (PDF)
