# Supplementary material for: Lignin Resists High-Intensity Electron Beam Irradiation
Source: Biomacromolecules. 2021 Sep 10;22(10):4365–72. doi: 10.1021/acs.biomac.1c00926 (PMC8512668; doi:10.1021/acs.biomac.1c00926)
Supplement: Supplementary file 1 — bm1c00926_si_001.pdf [file bm1c00926_si_001.pdf]

Supplementary information

Lignin resists high-intensity electron beam irradiation.

Oliver Sarosi[a], Irina Sulaeva[a], Elisabeth Fitz[a], Ivan Sumerskii[b], Markus Bacher[b], Antje Potthast[b]*

[a] Kompetenzzentrum Holz GmbH, Altenbergerstraße 69, A-4040 Linz

[b] Institute of Chemistry of Renewable Resources, Department of Chemistry, University of Natural Resources and Life Sciences, Konrad-Lorenz-Straße 24, A-3430 Tulln

*Phone: +43 1 47654-77412, 77471, e-Mail: antje.potthast@boku.ac.at

Keywords: milled wood lignin, kraft, lignosulfonate, radical, antioxidant, molar mass

Table of Contents

1. Raw materials for MWL 1
2. GPC data 2
3. HSQC NMR 2
  3.1. Crosspeaks and label assignment. 2
  3.2. Volume integrals of characteristic lignin bonds. 3
  3.3. HSQC-NMR spectra 4

1. Raw materials for MWL

Table 1: Chemical composition of wood used for milled wood lignin production.

Wood	*Fagus sylvatica* short-term storage (A)	*Fagus sylvatica* long-term storage (B)	*Eucalyptus grandis x urophylla* (C)
Dry content	55.2%	57.3%	51.6%
Acetone extractives	0.50%	0.98%	1.04%
Ash content	0.08%	0.33%	0.33%
Klason-lignin	21.9%	21.1%	20.2%
Acid soluble lignin	3.0%	3.5%	3.0%
Glucan	49.1%	48.5%	40.7%
Other carbohydrates	14.8%	22.6%	20.3%

2. GPC data

Table 2: Calculated statistical moments of lignin samples before and after irradiation from gel permeation chromatography.

Sample	M_n (kDa)	M_w (kDa)	M_z (kDa)	Đ
MWL A reference	2.1	14.6	77.3	6.9
MWL A 200	2.0	17.7	139.0	8.8
MWL B reference	26.8	608.6	5689.4	22.8
MWL B 200	28.8	538.2	5256.3	18.7
MWL C reference	3.0	16.5	67.7	5.5
MWL C 200	3.3	16.3	79.5	4.9
KL reference	0.5	1.4	5.5	3.1
KL 200	0.4	1.5	4.7	3.4
LS reference	1.1	8.0	41.6	7.2
LS 200	1.0	7.9	48.0	8.1

3. HSQC NMR

3.1. Crosspeaks and label assignment.

Table 3: Label, chemical shift and assignments of crosspeak signals in HSQC NMRs of all lignin samples.

**Crosspeak Label**	**H (ppm)**	**C (ppm)**	**Assignment**
A-β	3.05	53.5	$C_β$-$H_β$ in β-β‘ (resinol) substructures (A)
MeO	3.72	55.7	Methoxy groups
B-γ	4.09	61.7	$C_γ$-$H_γ$ in *p*-hydroxycinnamyl alcohol end groups (B)
C-γ	3.69	59.5	$C_γ$-$H_γ$ in β-O-4‘ substructures € and others
D-β	2.75	60.1	$C_β$-$H_β$ in β-1‘ (spriodienone) substructures (D)
X-5	3.31	63.8	C_5-H_5 in Xylopyranose
α-SA (α)	3.98	67.7	$C_α$-$H_α$ with a sulfonic acid group in α position
A-γ1	4.17	71.1	$C_{γ1}$-$H_{γ1}$ in β-β‘ (resinol) substructures (0)
A-γ2	3.79	71.0	$C_{γ2}$-$H_{γ2}$ in β-β‘ (resinol) substructures (0)
C-α	4.85	71.9	$C_α$-$H_α$ in β-O-4’ substructures €
X-2	3.01	72.7	C_2-H_2 in Xylopyranose
X-3	3.22	74.0	C_3-H_3 in Xylopyranose
X-4	3.54	75.7	C_4-H_4 in Xylopyranose
D-β‘	4.11	79.4	$C'_β$-$H'_β$ in β-1‘ (spriodienone) substructures (D)
α-SA (β)	4.92	79.5	$C_β$-$H_β$ with a sulfonic acid group in α position
C-β (G’)	4.48	80.6	$C_β$-$H_β$ in β-O-4’ substructures € linked to a G’ unit
D-α	5.06	81.1	$C_α$-$H_α$ in β-1‘ (spriodienone) substructures (D)
BE-α	4.74	81.4	$C_α$-$H_α$ in benzyl ether LCC structures
C‘-β	5.21	83.1	$C_β$-$H_β$ in β-O-4’ substructures € with oxidation in α-position
C-β (G, erytho)	4.3	83.5	$C_β$-$H_β$ in β-O-4’ substructures € linked to a G unit erythron
C-β (G, threo)	4.3	83.1	$C_β$-$H_β$ in β-O-4’ substructures € linked to a G unit threo
A-α	4.63	85.1	$C_α$-$H_α$ in β-β‘ (resinol) subsrtructures (A)

Table 3 continued.

Crosspeak Label	H (ppm)	C (ppm)	Assignment
C-β (S)	4.11	86.0	$C_β$-$H_β$ in β-O-4′ substructures (C) linked to a S unit
D-α‘	4.33	86.6	$C_{α'}$-$H_{α'}$ in β-1‘ (spriodienone) substructures (D)
E-α	5.44	86.9	$C_α$-$H_α$ in β-5‘ (phenylcoumaran) substructures (E)
X-1	4.27	101.7	C_1-H_1 in Xylopyranose
(1—>4)-β-D-Manp	4.66	101.6	C_1-H_1 in β-D-Mannose with a 1—4-glycosidic linkage.
S-2,6	6.64	103.6	C_2-H_2 and C_6-H_6 in syringyl units (S)
S'-2,6	7.27	106.3	C_2-H_2 and C_6-H_6 in oxidized syringyl units (S)
G-2	6.9	111.2	C_2-H_2 in guaiacyl units (G)
G'-2	7.45	111.5	C_2-H_2 in oxidized guaiacyl units (G)
D-2‘	6.25	113.5	$C_{2'}$-$H_{2'}$ in β-1‘ (spirodienone) substructures (D)
G-5	6.69	114.8	C_5-H_5 in guaiacyl units (G)
G'-5	6.92	114.9	C_5-H_5 in oxidized guaiacyl units (G)
D-6‘	6.09	118.8	$C_{6'}$-$H_{6'}$ in β-1‘ (spirodienone) substructures (D)
G-6	6.75	120.0	C_6-H_6 in guaiacyl units (G)
G‘-6	7.55	123.2	C_6-H_6 in oxidized guaiacyl units (G)
Stilbene (α+β)	6.99	126.3	-
F-β	6.78	126.3	$C_β$-$H_β$ in p-hydroxycinnamyl aldehyde end groups (F)
H-2,6	7.18	127.8	C_2-H_2 and C_6-H_6 in *p*-hydroxyphenyl units (H)
B-β	6.45	128.4	$C_β$-$H_β$ in *p*-hydroxycinnamyl alcohol end groups
B-α	6.33	130.0	$C_α$-$H_α$ in *p*-hydroxycinnamyl alcohol end groups

3.2. Volume integrals of characteristic lignin bonds.

Table 4: HSQC volume integral intensity of characteristic structural motifs of all investigated lignin samples before and after EBI at 200 kGy. Intensities are normalized to lignin mass and representative C9 unit signals.

Crosspeak Label	MWL A ref (%)	MWL A 200 (%)	MWL B ref (%)	MWL B 200 (%)	MWL C ref (%)	MWL C 200 (%)	KL ref (%)	KL 200 (%)	SL ref (%)	SL 200 (%)
A-β	11.3	11.5	15.6	15.9	14.8	14.5	4.9	5.0	6.9	6.8
MeO	559.3	559.7	601.9	583.8	598.6	602.2	462.2	453.9	762.7	759.4
B-γ	-	-	-	-	-	-	1.0	0.8	-	-
C-γ	63.3	64.3	62.1	61.9	67.2	61.6	-	-	-	-
D-β	0.6	0.8	-	-	-	-	-	-	-	-
X-5	-	-	35.7	34.8	-	-	-	-	-	-
α-SA (α)	-	-	-	-	-	-	-	-	0.8	0.9
A-γ1	10.5	10.5	14.4	13.9	11.4	12.6	5.5	5.6	10.3	10.2
A-γ2	12.2	11.1	17.7	16.0	14.5	13.9	6.4	6.6	8.0	7.5
C-α	63.8	64.5	37.5	36.5	53.7	54.2	3.6	3.6	3.2	3.3
X-2	2.3	2.0	1.4	1.3	1.1	1.2	1.9	2.2	-	-
X-3	2.9	3.3	1.8	1.8	1.8	2.2	1.9	2.2	-	-
X-4	7.3	7.7	3.2	2.8	4.1	3.9	2.3	2.7	-	-

Table 4: continued.

Crosspeak Label	MWL A ref (%)	MWL A 200 (%)	MWL B ref (%)	MWL B 200 (%)	MWL C ref (%)	MWL C 200 (%)	KL ref (%)	KL 200 (%)	SL ref (%)	SL 200 (%)
D-β‘	0.6	0.7	-	-	-	-	-	-	-	-
α-SA (β)	-	-	-	-	-	-	-	-	2.9	3.4
C-β (G‘)	2.5	3.0	16.2	15.5	4.3	4.6	-	-	2.2	2.2
D-α	2.4	2.6	0.5	0.5	0.7	0.5	-	-	-	-
BE-α	-	-	-	-	-	-	2.1	1.9	10.0	10.4
C‘-β	1.7	1.8	1.9	1.8	1.7	1.8	-	-	0.8	0.7
C-β (G, erytho+threo)	15.7	15.4	14.2	14.3	11.6	10.9	0.6	0.7	-	-
A-α	16.3	16.7	20.4	19.4	17.9	18.6	5.6	5.6	11.7	10.9
C-β (S)	34.9	35.9	32.6	31.9	31.9	32.1	0.7	0.6	0.6	0.6
D-α‘	4.1	4.5	4.5	4.4	3.3	4.3	1.3	1.4	2.4	2.3
E-α	3.5	3.3	4.0	3.8	2.8	3.1	0.2	0.2	0.2	0.3
X-1	-	-	-	-	-	-	1.1	1.3	-	-
(1—>4)-β-D-Manp	-	-	2.5	2.7	-	-	-	-	-	-
S-2,6	113.7	111.8	112.4	109.4	112.8	112.6	149.9	152.3	170.2	169.3
S'-2,6	11.1	12.9	9.5	11.1	11.2	11.4	4.8	4.5	21.4	22.0
G-2	36.2	36.2	37.4	38.0	36.0	35.6	22.7	21.6	-	-
G'-2	1.4	1.5	1.6	1.7	2.0	2.4	-	-	4.2	4.3
D-2‘	1.2	1.3	-	-	-	-	-	-	-	-
G-5	21.7	22.8	29.9	28.5	33.0	36.0	28.1	29.0	87.5	87.6
G'-5	14.9	16.5	15.6	15.5	15.6	17.3	-	-	-	-
D-6‘	1.0	1.2	-	-	-	-	-	-	-	-
G-6	29.1	32.6	36.7	36.6	29.2	37.3	28.5	27.8	19.9	20.1
G‘-6	0.9	1.0	0.9	0.9	2.4	2.5	0.9	1.0	1.7	1.7
Stilbene (α+β)	-	-	-	-	-	-	3.9	4.1	-	-
F-β	1.3	3.2	2.2	3.1	1.3	4.4	-	-	-	-
H-2,6	2.5	3	-	-	3.6	8.8	-	-	8.9	8.9
B-β	4.6	4.6	-	-	-	-	-	-	-	-
B-α	4	2	-	-	-	-	-	-	-	-

3.3. HSQC-NMR spectra

Figures 1-10 show the HSQC-NMR spectra of all tested lignin samples. Reference and irradiated lignin variant are compared based on an overview and zoom cuts for linkage and aromatic region, respectively.

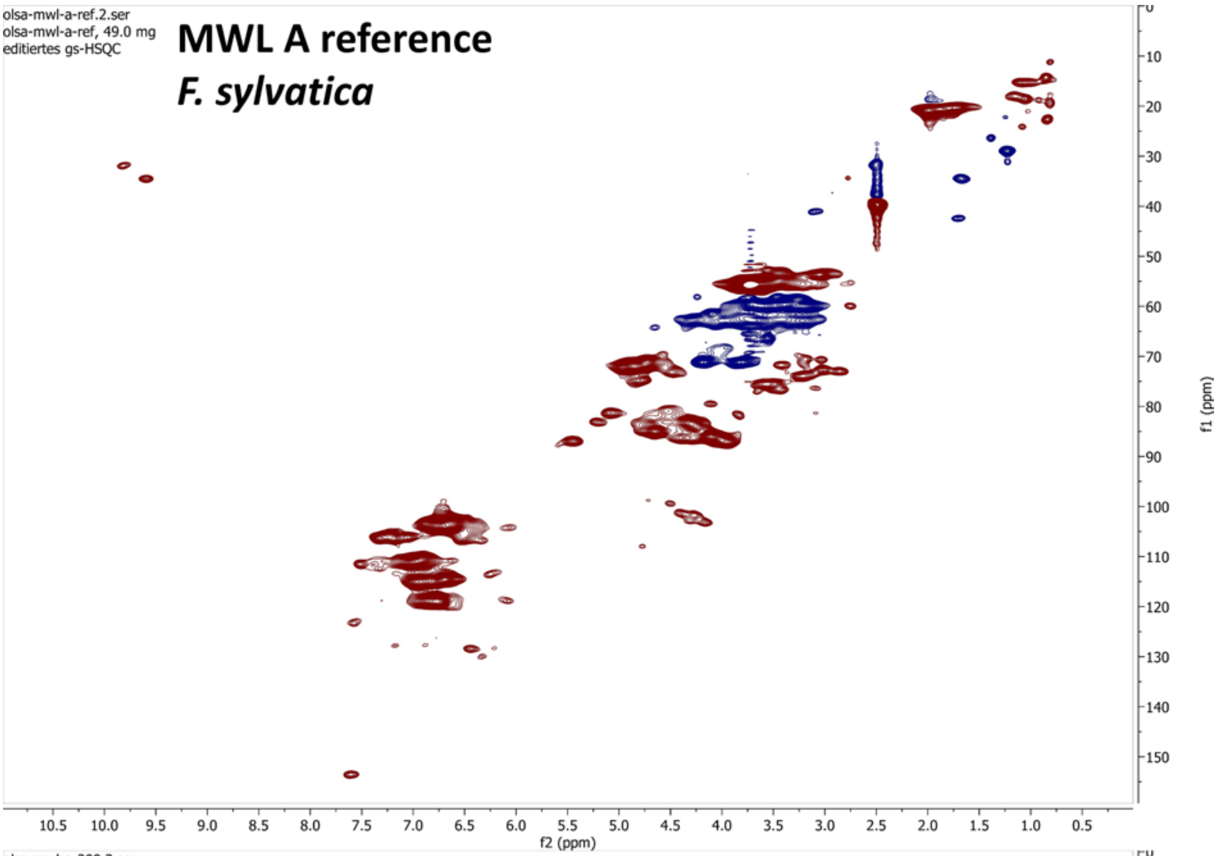

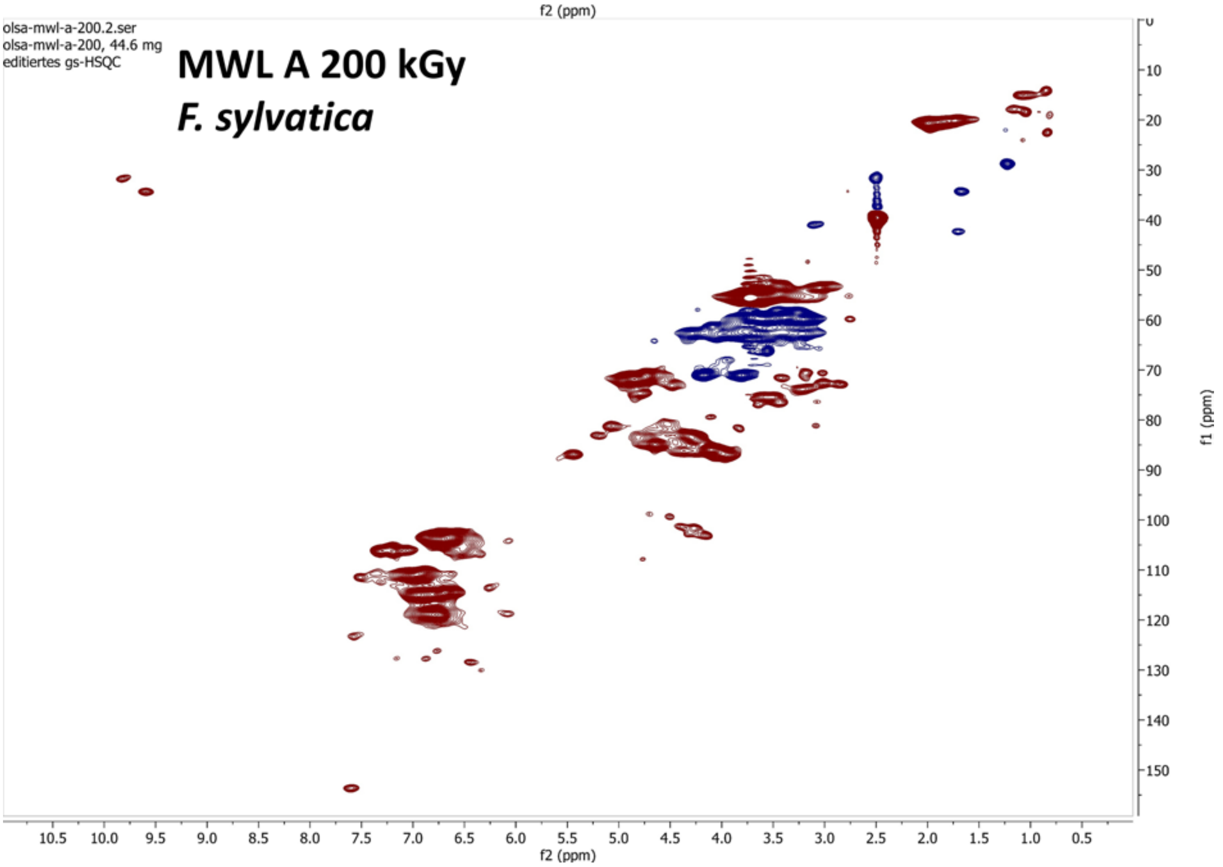

Figure 1: MWL A overview.

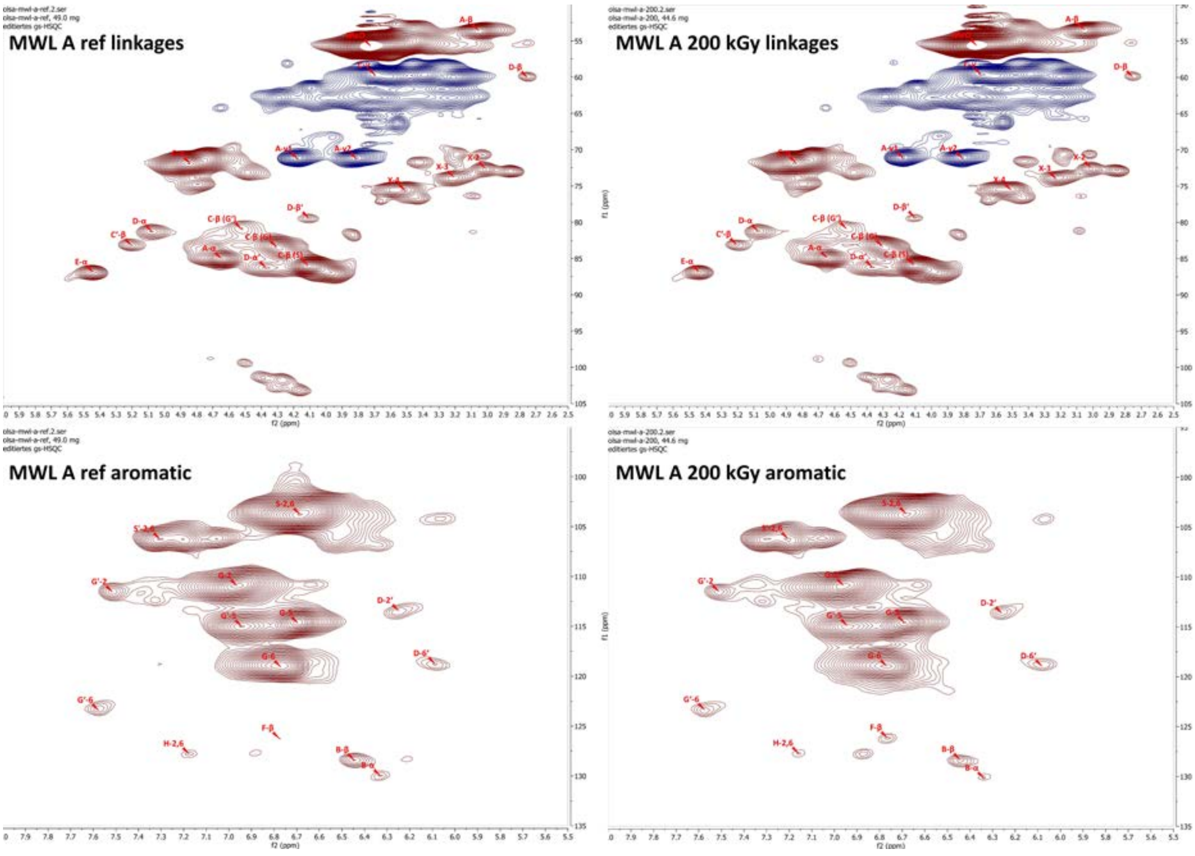

Figure 2: MWL A zoomed regions.

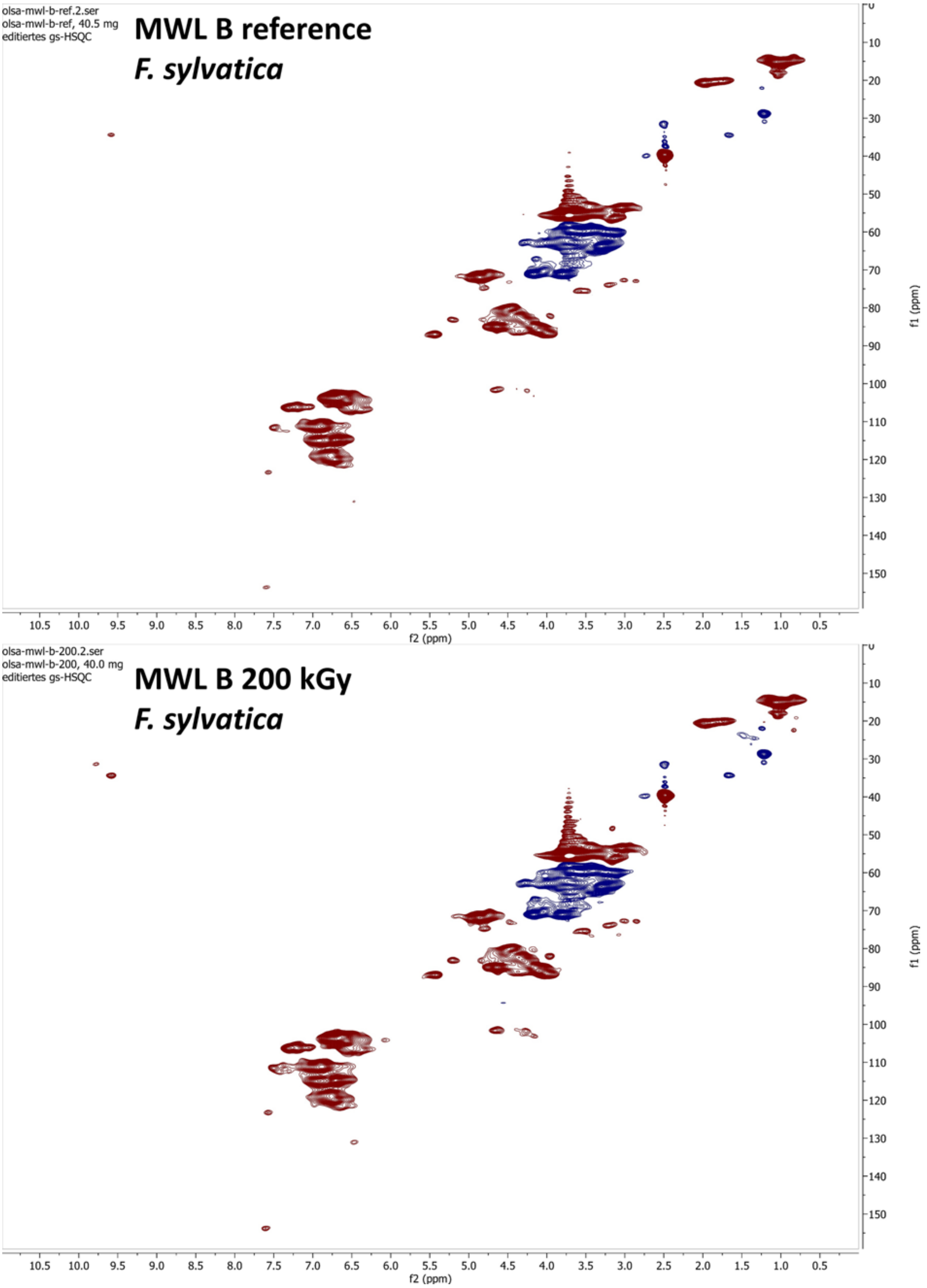

Figure 3 MWL B overview.

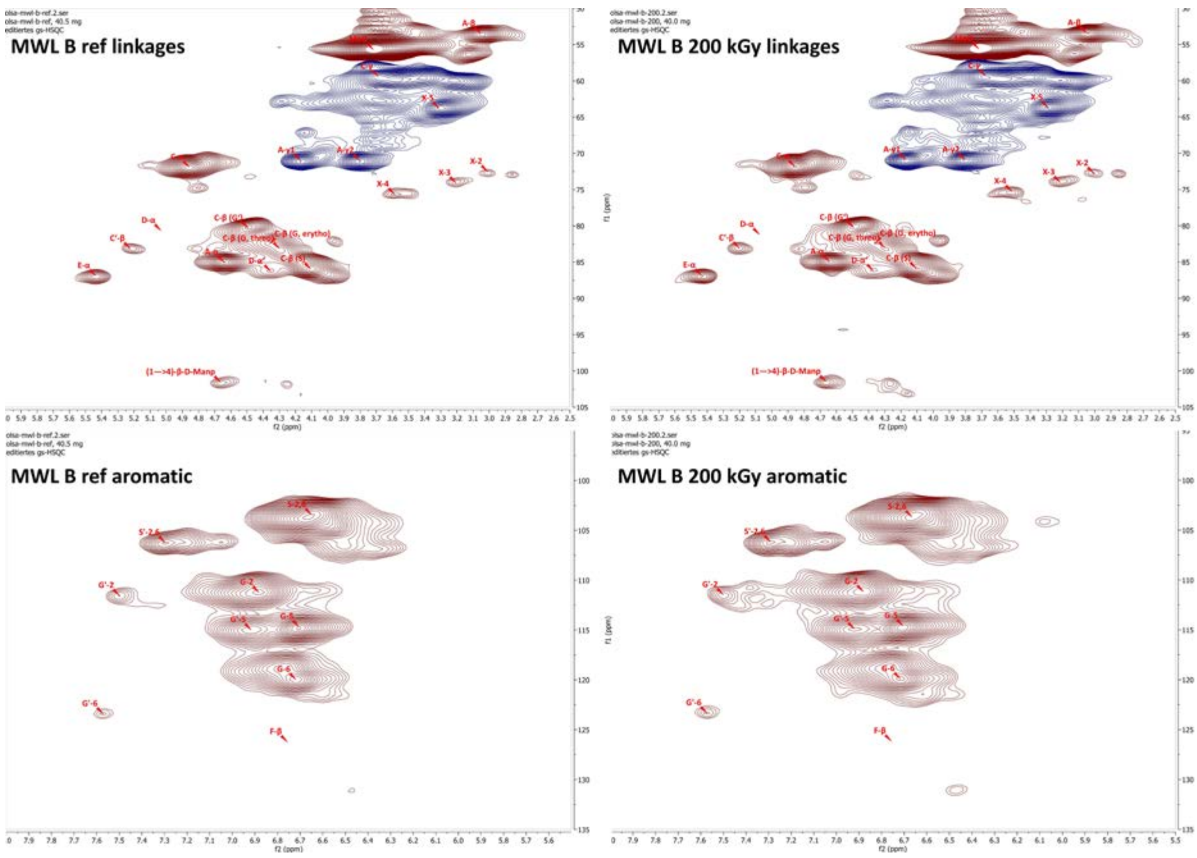

Figure 4: MWL B zoomed regions.

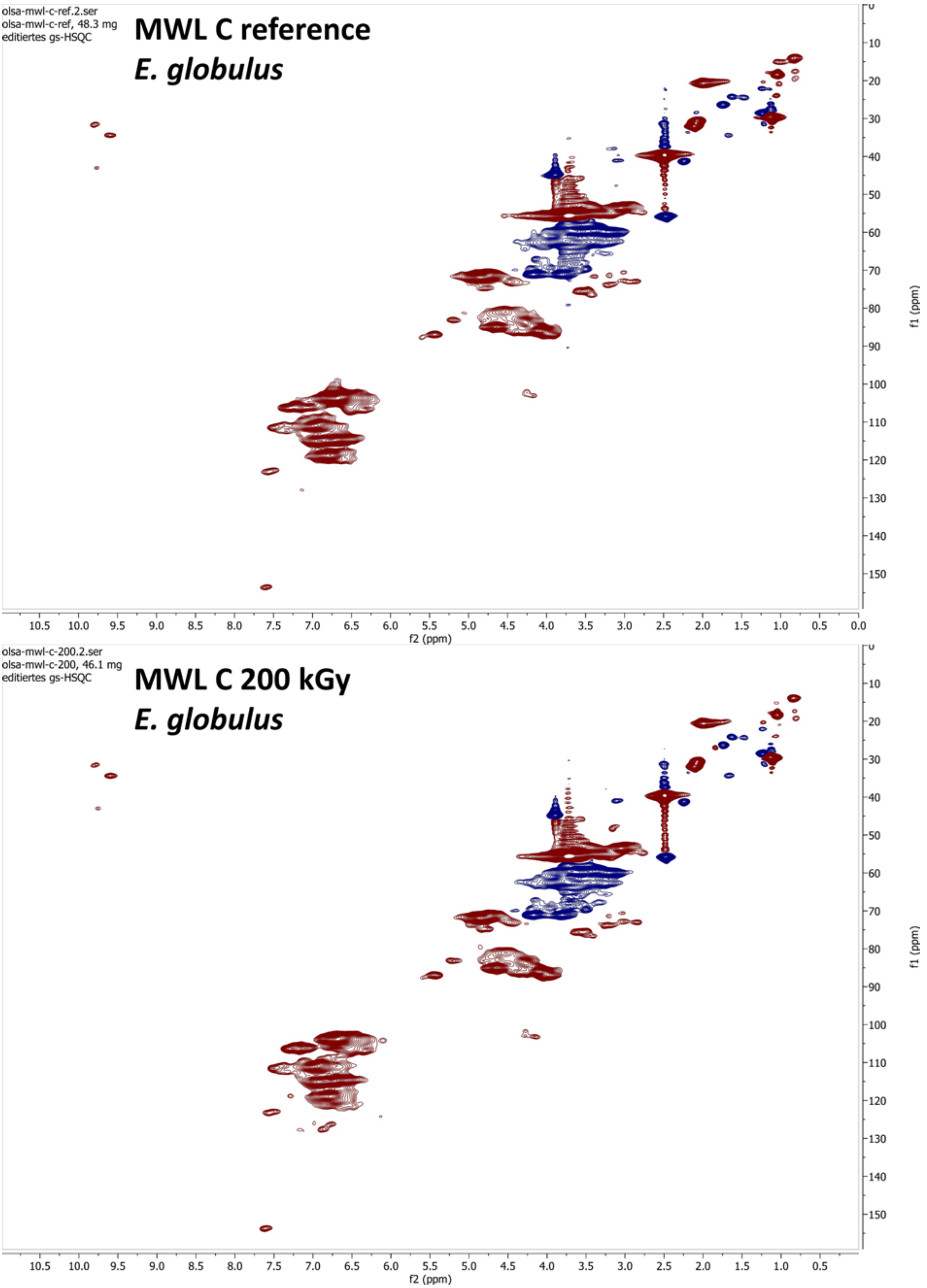

Figure 5: MWL C overview.

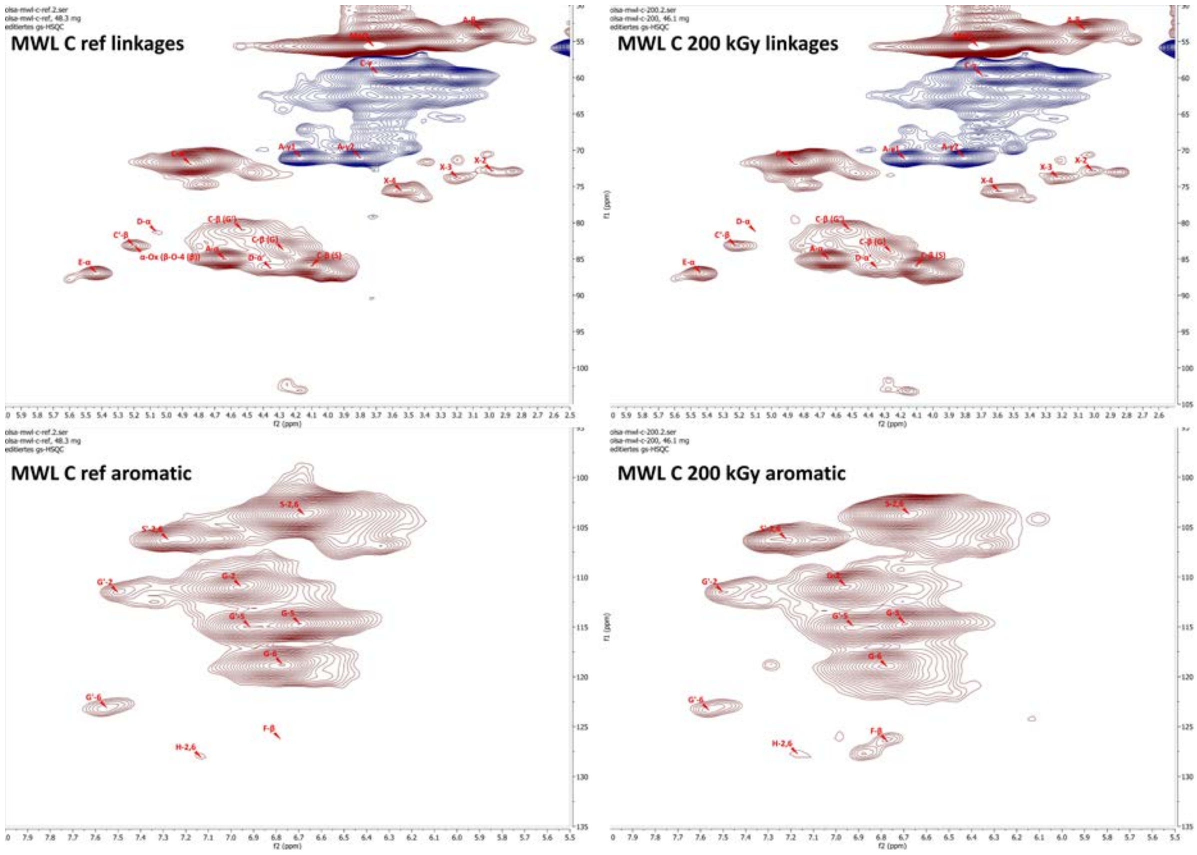

Figure 6: MWL C zoomed regions.

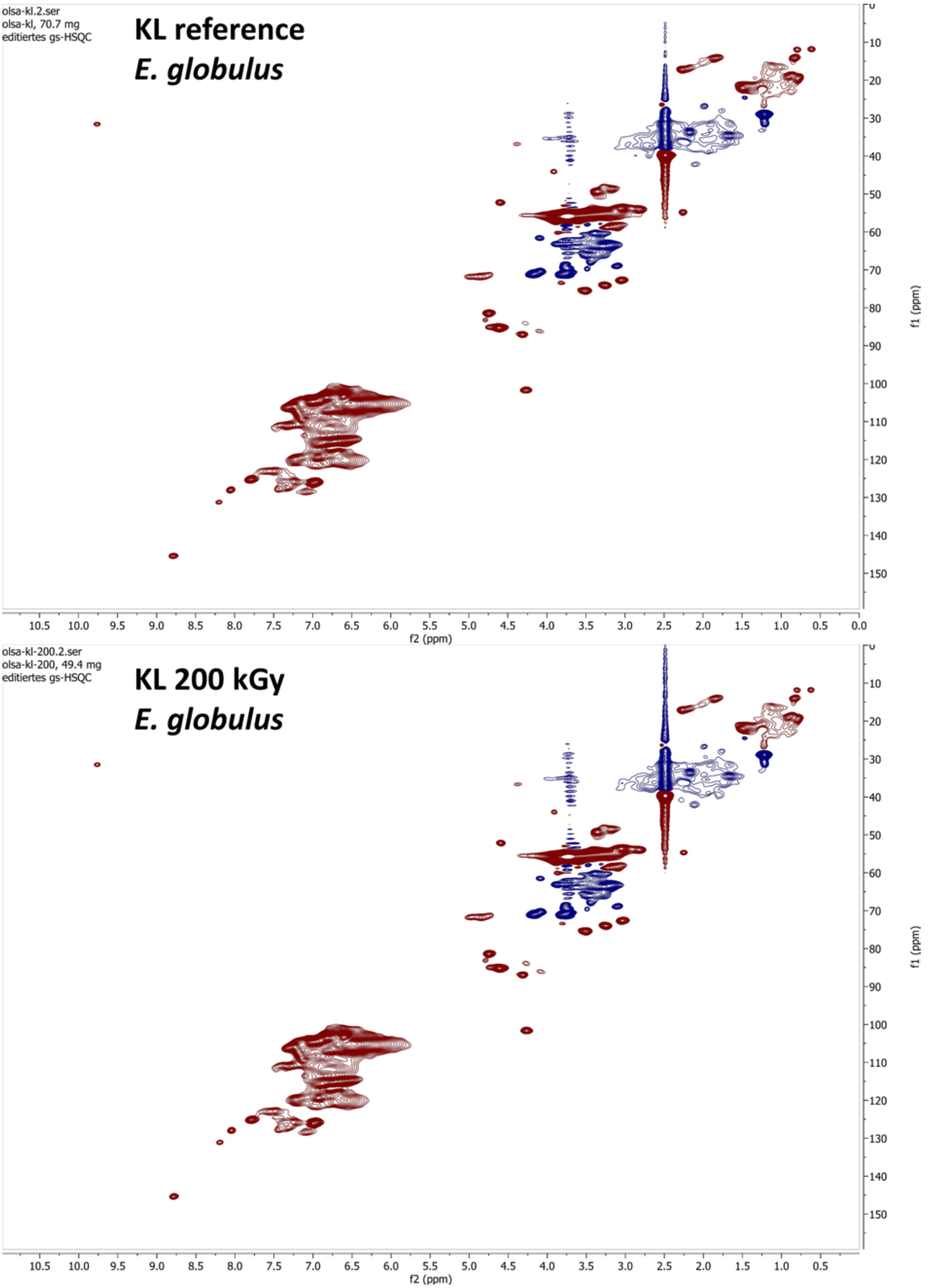

Figure 7: KL overview.

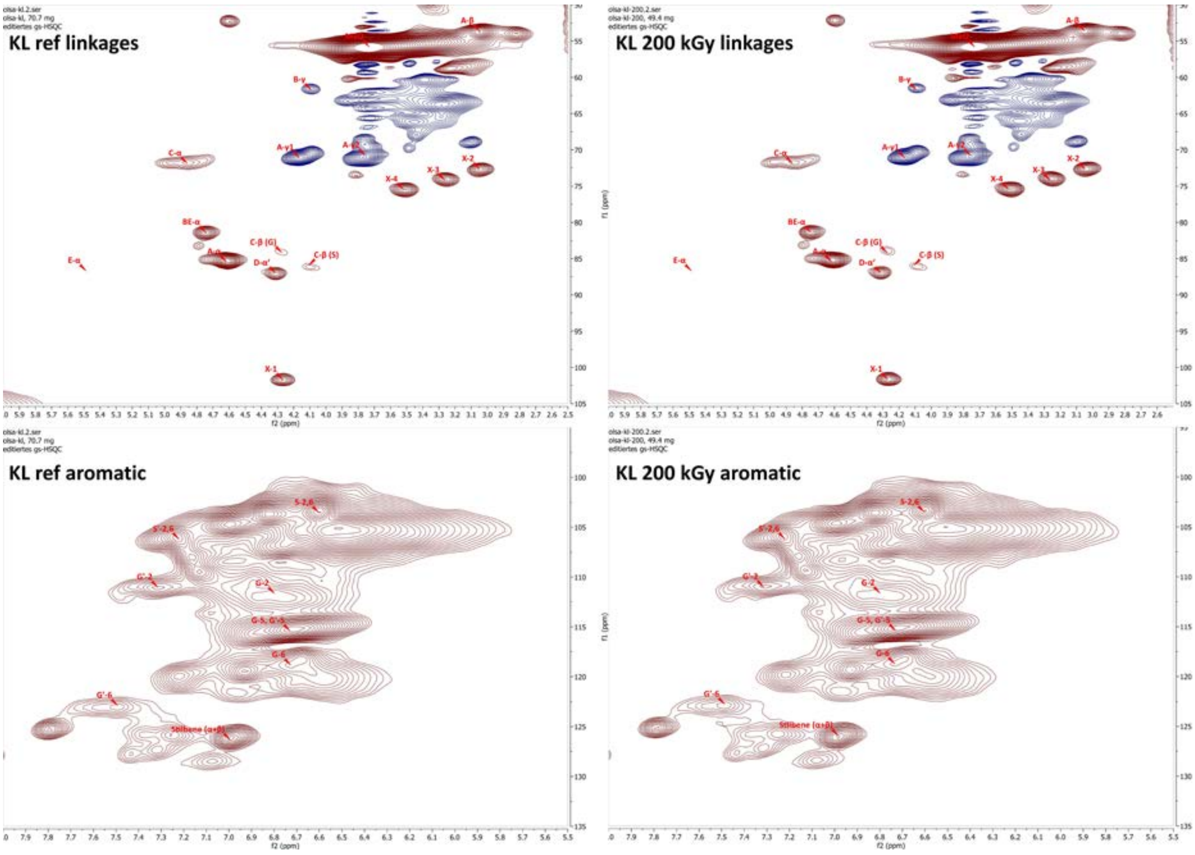

Figure 8: KL zoomed sections.

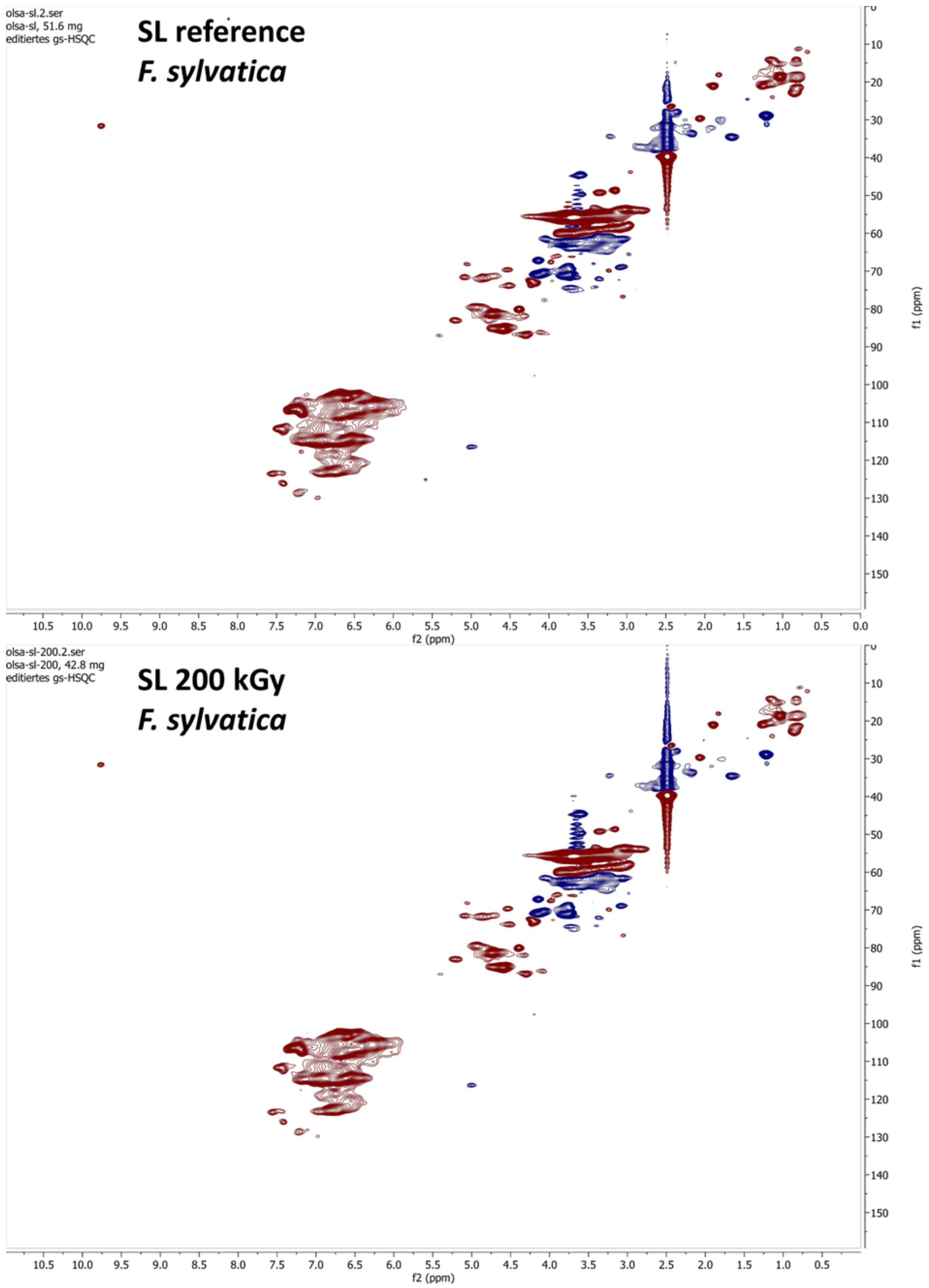

Figure 9: SL overview.

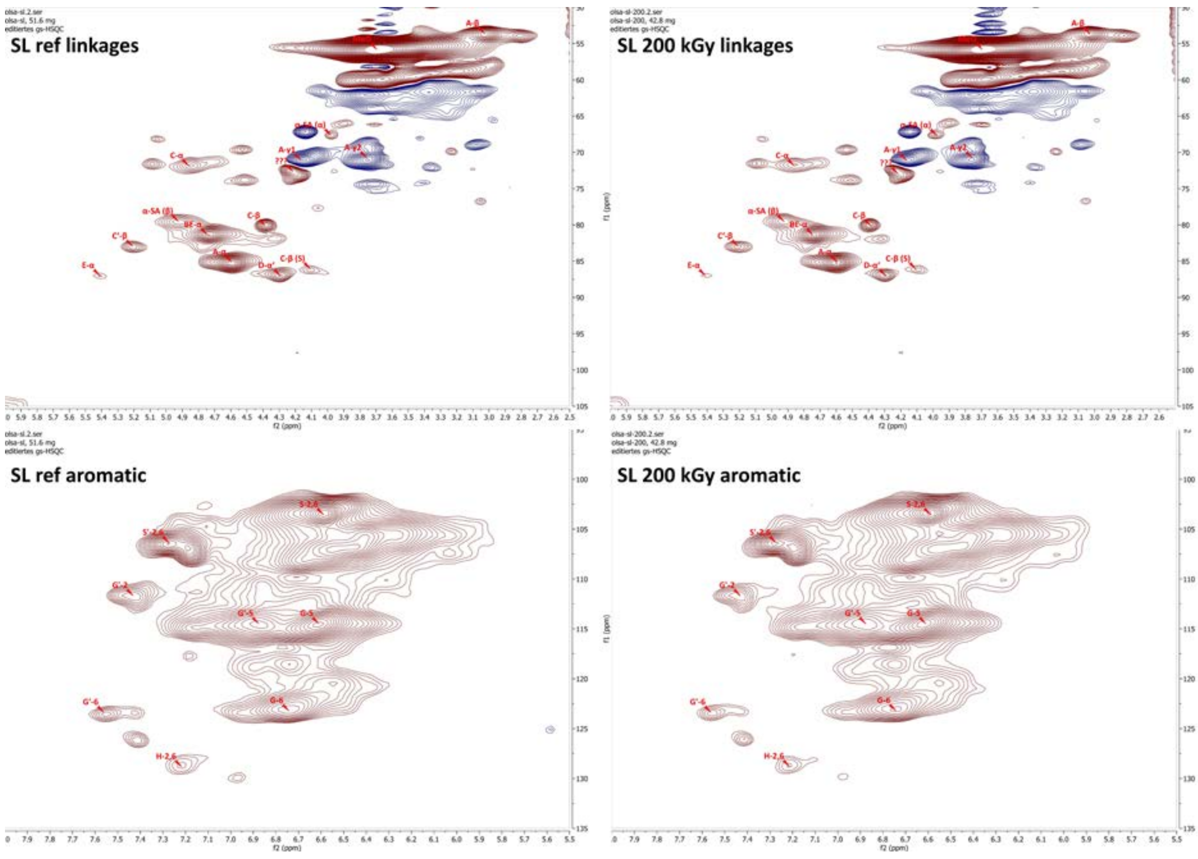

Figure 10: SL zoomed regions.
